# Awareness and knowledge of undergraduate dental students about the signs and symptoms of Corona viral infection (COVID-19), and the required infection control measures to prevent its spread

**DOI:** 10.1186/s42269-021-00494-1

**Published:** 2021-02-01

**Authors:** Rasha F. Sharaf, Nihal Kabel

**Affiliations:** 1 Orthodontics and Pediatric Dentistry Department, National Research Centre, Cairo, Egypt; 2 Pediatric Dentistry and Community Dentistry Department, Misr University for Science and Technology, Giza, Egypt

**Keywords:** COVID-19, Dental students, Awareness, Infection control

## Abstract

**Background:**

Coronavirus disease (COVID-19) is considered a highly contagious disease with flu-like symptoms and causing relatively high level of death. It can be transmitted from a person to another through droplets and that makes the dentists at high risk of infection. Therefore, the aim of the current study was to assess the awareness and knowledge of dental students about the signs and symptoms of Coronavirus disease (COVID-19) and to evaluate their awareness about the required infection control measures during the dental treatment to control the spread of the disease. A questionnaire was formed using Google forms and distributed among dental students and interns in different universities in Cairo, Egypt. Questions were about signs and symptoms of COVID-19, attitude of the dentists toward dental treatment of suspected patients and the required personal protective equipment (PPE) and infection control measures at the dental clinic.

**Results:**

The majority of the participants strongly agreed/agreed that COVID-19 is a highly dangerous disease, Participants reported that the most common symptom is difficulty in breathing (89.4%) followed by fever (84.4%). Face shield was the most recommended personal protective equipment (PPE) during dental treatment (98.6%) followed by disposable gown (96.3%). The majority of participants (84.8%) recommended using 70% ethyl alcohol as the first method to disinfect surfaces in between dental visits, followed by sodium hypochlorite.

**Conclusions:**

Dental students and interns in Cairo, Egypt, have good knowledge and awareness about COVID-19 and the necessary precautions required to provide adequate dental treatment for the patients during the pandemic COVID-19; however, the importance of infection control should be highlighted for both clinical and preclinical dental students, to provide safe dental treatment to the patients as well as protection of the dentists and healthcare workers.

## Background

Coronavirus disease (COVID-19) appeared for the first time in Wuhan, China, in 2019; then, it started to spread through the whole world causing fear and anxiety among all nations, being highly contagious and causing relatively high level of death. The World Health Organization (WHO) considered it a pandemic disease on March 2020 (Chowdhury et al. 2020; Lie et al. 2020).

The virus was found to be a single-stranded (SS) ribonucleic acid (RNA), enveloped virus (Yip et al. 2020), which invades the upper respiratory tract causing severe infection, and it causes flu-like symptoms like high grade fever, fatigue, dry cough and difficulty in breathing. Also, the chest computed tomography (CT) shows abnormal images. Other symptoms are less common to appear like headache, sputum production, dyspnea, loss of taste and smell, and diarrhea (Kumar et al. 2020).

The average incubation period of COVID-19 ranges from 4 to 14 days; however, some reports stated that it may extend to 24 days. Elderly patients with debilitating diseases are considered to be at very high risk getting infected compared to healthy, young aged patients (Mueller et al. 2020; Duruk et al. 2020).

The disease can be transmitted from one person to another through airborne droplets; the virus can also stay live on different surfaces for few hours or up to several days, depending on the type of surface, humidity and the temperature (Singhal 2020).

COVID-19 can be transmitted through hands, nasal droplets and surface contact. It can also be transmitted through saliva and blood such as Hepatitis B virus (HBV), Hepatitis C virus (HCV) and human immunodeficiency virus infection/acquired immune deficiency syndrome (HIV/AIDS) that makes dentists at the top of the hierarchy of people at risk of being infected due to their close proximity to the patients during performing the dental treatment (Jayaweera et al. 2020).

What makes the dentists at high risk of being infected not only being in close contact with their patients but also being exposed to the aerosols, droplets and saliva splashing out of the patients’ mouth, so the dentists are considered not only at high risk of being infected but also at high risk of spreading the infection to their families and other healthy patients (Nejatidanesh et al. 2013).

The American Dental Association (ADA) stated some precautions to decrease the risk of transmission of the infection, in addition to the standard infection control protocol, which include recording the temperature for every patient, rinsing with 1% hydrogen peroxide before any dental procedures, highlighting the importance of using rubber dam and high volume suction during dental procedures and using of disinfectants in cleaning of all surfaces, chairs and door handles (Meng et al. 2020).

In the current situation, many of the graduating dental students feel fearful of being infected with COVID-19 or transmitting the disease to others during their dental practice; this fear and anxiety may be due to being unaware about the necessary precautions and infection control procedures that should be followed to keep them safe and decrease the risk of being infected and that highlights the importance of being aware with the recent guidelines (Meng et al. 2020; Suryakumari et al. 2020).

Therefore, a questionnaire-based study was conducted to assess the awareness and knowledge of dental students about the signs and symptoms of Corona viral infection (COVID-19) and to evaluate their awareness about the required infection control measures that should be performed during the dental treatment to control the spread of the disease.

## Methods

Before starting that questionnaire, the idea was discussed and the content adequacy of the questionnaire was examined by experts in medical science, microbiology, infection control, healthcare quality, biostatistics and quality management, to assess the accuracy and clearance of the questions and their validation. The experts modified 3 questions and added some changes in the answers. Based on the comments made by these experts, 3 questions were modified and 2 questions were added.

The questionnaire was approved by the Ethics Committee at the National Research Centre of Egypt and recorded by number 20084. A pilot study was performed, and the questionnaire was distributed among 40 dental students to check whether it was clear and understandable or not. The feedback was that all questions were clear and easy to understand, and then, the final online questionnaire was formed using Google forms.

The present study is a cross-sectional study, conducted using an online survey in the duration from 5 April till 20 April 2020 using Google forms (www.googleforms.com), a well-constructed and validated questionnaire was formed and its link was sent to dental students in the 3rd, 4th, 5th year and interns in different Universities in Cairo, Egypt, and circulated through the social media.

The questionnaire was formed of 15 close-ended questions. At the beginning of the questionnaire, there was an introduction to explain the aim of the survey and that no personal data were required; also, it was mentioned that the decision of participation in that survey was completely voluntary and will not affect the students and their courses.

If the participants agree to take part in the survey, they had to answer all the questions and at any time they feel they want to quit, there was no problem at all and any form with incomplete answers was excluded.

The first part of the questionnaire started with asking if the participant was male or female and identifying the studying year at Faculty of Oral and Dental Medicine either 3rd, 4th, 5th or intern. The second part of the questions was related to the different signs and symptoms of Corona viral infection (COVID-19) to check the awareness of the dental students about these signs and symptoms and the attitude of the dental students at the dental clinic toward suspected infected patients. The third part included different questions about personal protective equipment (PPE) of the dentists and the required infection control measures at the dental clinic for protection of the dentist and the patient.

### Sample size calculation

**Fig. 1:**
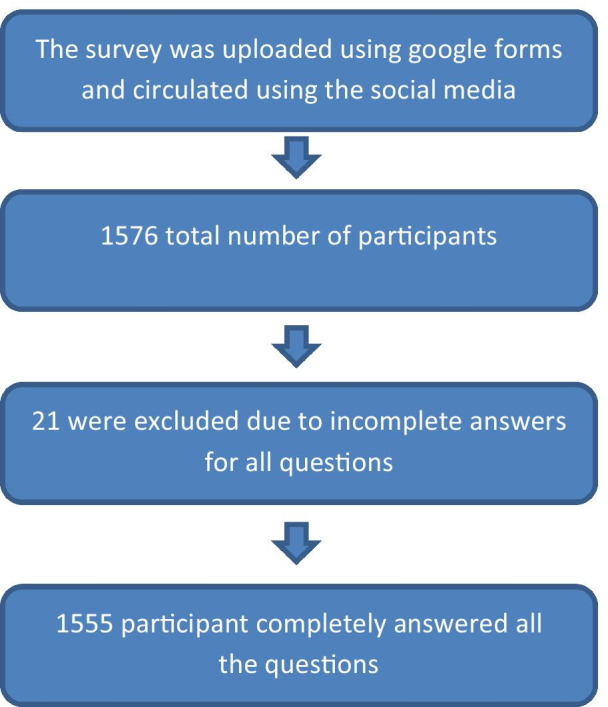
Flowchart showing recruitment of the participants

This power analysis used percentage of knowledge about COVID-19 as the primary outcome. The percentage of knowledge was estimated to be 50%, using alpha (*α*) level of (5%), acceptable margin of error = 3%; the minimum estimated sample size was 1066 subjects. Sample size calculation was performed using Epi Info 7.2.2.2. The number of the participants of dental students and intern who shared in the current survey exceeded the required sample size, as 1576 dental student and intern participated in the survey, after exclusion of incomplete forms, 1555 was the total number of completely answered forms (Fig. 1).

### Statistical analysis

Qualitative data were presented as frequencies and percentages. Statistical analysis was performed with IBM SPSS Statistics for Windows, Version 23.0. Armonk, NY: IBM Corp.

### Results

#### Personal information

The present study was conducted on 1555 dental students and interns: 969 females (63%) and 586 males (37%).

**Fig. 2:**
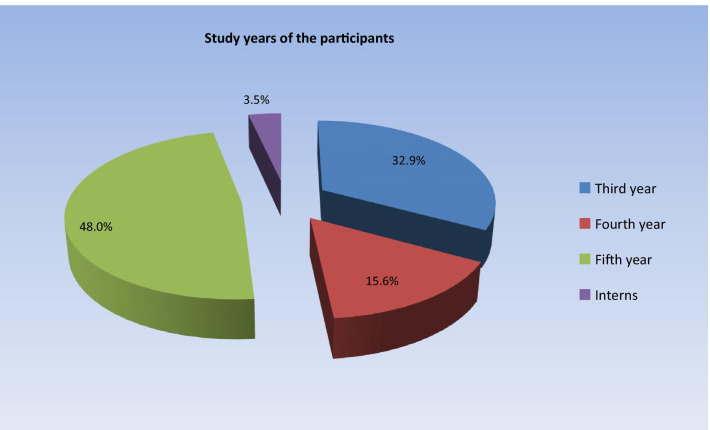
Pie chart representing the distribution of study years among study participants

Nearly half of the subjects were fifth-year (last year) students followed by third-year students comprising about one third of the participants then fourth-year students (15.6%) and (3.5%) interns (Fig. 2).

#### Knowledge and attitudes towards COVID-19

The majority of the participants strongly agreed/agreed that COVID-19 is a highly dangerous disease, while only 0.7% did not know if it is dangerous or not. Participants reported that the most common symptom they can suspect COVID-19 patients is difficulty in breathing (89.4%) followed by fever (84.4%), while only 7.2% of the participants thought that wet cough is a symptom of COVID-19. The majority of participants agreed that high-speed hand piece can increase the risk of transmission of COVID-19. Almost all participants agreed that saliva can transmit COVID-19 (97.2%) (Table 1).

**Table 1:** Frequencies (*n*) and percentages (%) for responses to questions regarding knowledge and attitudes toward COVID-19 (*n* = 1555)

Knowledge and attitudes toward COVID-19	*n*	%
COVID-19 is a highly dangerous disease		
Strongly agree	1002	64.4
Agree	505	32.5
I don’t know	11	0.7
Disagree	32	2.1
Strongly disagree	5	0.3
Patient is suspected to be COVID-19 positive if he is suffering from		
Fever	1312	84.4
Dry cough	1240	79.7
Wet cough	112	7.2
Loss of taste and smell	953	61.3
Pain all over the body	1030	66.2
Difficulty in breathing	1390	89.4
Sore throat	796	51.2
Sneezing	404	26.0
Saliva can transmit COVID-19	1512	97.2
Using a high-speed hand piece in performing the dental treatment can increase the risk of transmission of COVID-19 to the dentist and in between the patients		
Agree	1280	82.3
Neutral	98	6.3
Disagree	177	11.4
If screening for the dental patients showed a patient with fever and suspected to be a COVID-19 positive patient; you will		
Accept to perform the dental treatment for him/her	194	12.5
Postpone the dental treatment to another visit	812	52.2
Refer the patient to another dentist who is more experienced in infection control	549	35.3

Regarding performing dental treatment for a suspected COVID-19 positive patient, almost half of participants preferred to postpone the dental treatment to another visit when seeing a patient suspected to be COVID-19 positive (Table 1).

#### Use of rubber dam

The majority of participants (70.9%) recommended and highly recommended using rubber dam during dental treatment to reduce the risk of COVID-19 transmission. The most common dental procedure that participants recommend using rubber dam with was endodontic treatment followed by restorative procedures and then pulpotomy for children, while the least recommended dental procedure was crown preparation (Table 2).

**Table 2:** Frequencies (*n*) and percentages (%) for answers regarding the use of rubber dam

Using rubber dam	*n*	%
Do you recommend using rubber dam during dental treatment to decrease the risk of transmission of COVID-19?		
Highly recommend	970	62.4
Recommend	132	8.5
Don’t recommend	25	1.6
Highly don’t recommend	428	27.5
Which dental procedures do you recommend using rubber dam with?		
Endodontic treatment	1414	90.9
Restorative procedures	1166	75.0
Crown preparation	540	34.7
Pulpotomy for children	884	56.8

#### Protection against COVID-19

The majority of participants (84.8%) recommended using ethyl alcohol 70% as the first method to disinfect surfaces in between dental visits, followed by sodium hypochlorite. The majority (88.4%) also recommended the use of N95 mask as better protection against COVID-19 transmission compared with surgical mask. Percentage of participants who thought that surgical masks provide better protection for persons in front of the ones wearing it was 56.3% compared with 43.7% of participants who thought it has better protection for the person wearing it (Table 3).

**Table 3:** Frequencies (*n*) and percentages (%) for answers regarding protection against COVID-19

Protection against COVID-19	*n*	%
Disinfection of surfaces in between the patients should be done using		
Sodium hypochlorite	962	61.9
Dettol	284	18.3
Ethyl alcohol 70%	1318	84.8
Methyl alcohol 70%	226	14.5
Which type of mask do you prefer to wear for better protection during performing dental treatment?		
N95	1374	88.4
Surgical mask	181	11.6
Surgical masks provide better protection for the person		
Wearing it	680	43.7
In front of the one wearing it	875	56.3

#### Instruments recommended to be used during emergency dental treatment

Hand instruments were the most recommended instruments to be used during emergency dental treatment (68%) followed by high-speed hand piece (62.9%), while low-speed hand piece was the least recommended instrument (61.7%) (Table 4).

**Table 4:** Frequencies (*n*) and percentages (%) for answers regarding instruments used during emergency dental treatment during COVID-19 pandemic

Instrument	Highly recommend		Recommend		Don’t recommend		Highly don’t recommend	
	*n*	%	*n*	%	*n*	%	*n*	%
Hand instrument	679	43.7	378	24.3	450	28.9	48	3.1
Low-speed hand piece	192	12.3	768	49.4	534	34.3	61	3.9
High-speed hand piece	510	32.8	468	30.1	378	24.3	199	12.8

#### Difference between the regular high-speed hand piece and the high-speed hand piece with anti-retraction valve

Only 28% (*n* = 436) of the participants reported they know the difference between high-speed hand piece and the high-speed hand piece with anti-retraction valve.

#### Personal protective equipment (PPE)

**Fig. 3:**
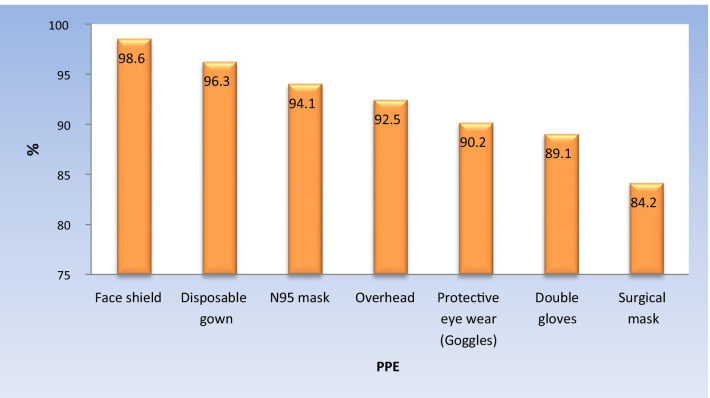
Bar chart representing distribution of important personal protective equipment (PPE)

Face shield was the most recommended PPE equipment to be used during dental treatment (98.6%) followed by disposable gown (96.3%), while surgical mask was the least recommended equipment (84.2%) (Fig. 3, Table 5).

**Table 5:** Frequencies (*n*) and percentages (%) for important PPE items that help dentists feel safe during dental treatment in the time of COVID-19 pandemic

Equipment	Very important		Important		Neutral		Not important	
	*n*	%	*n*	%	*n*	%	*n*	%
Double gloves	1110	71.4	276	17.7	120	7.7	49	3.2
Surgical mask	780	50.2	528	34.0	174	11.2	73	4.7
N95 mask	1350	86.8	114	7.3	48	3.1	43	2.8
Disposable gown	1308	84.1	189	12.2	56	3.6	2	0.1
Overhead	1163	74.8	275	17.7	114	7.3	3	0.2
Face shield	1318	84.8	214	13.8	23	1.5	0	0.0
Protective eye wear (Goggles)	1099	70.7	303	19.5	118	7.6	35	2.3

## Discussion

The present study reported the awareness and knowledge of the undergraduate dental students and interns about the signs and symptoms of the Corona viral infection (COVID-19), and the required infection control measures to be taken during the current viral outbreak.

Questionnaire-based studies are proven as highly effective for gathering information regarding the awareness and knowledge of the dental students about COVID-19 and the protection equipment preferences of participants; however, careful data collection and interpretation is required (Sengupta et al. 2020).

The questionnaire used in the present study collected information objectively, through an online site for a period of two weeks. High level of participation was noted (*n* = 1555), which exceeded the calculated sample size (*n* = 1066) and increased the validity and reliability of the obtained results. The high level of participation possibly due to the fact that COVID-19 is a current issue which the dentists and dental students are worried about and interested in, as they do not know precisely the appropriate reactions and actions needed to be taken.

Since it has been approved that the primary route for transmission of COVID-19 is through air droplets and aerosols (Singhal 2020), the transmission of COVID-19 poses a risk for people who come in close contact with an infected individual, and the risk is greater among those who are in close proximity to or work near the patient. The distance between the working field and the dentist is approx. 35–40 cm, and certain procedures can be very time-consuming, thereby increasing the risk of dentists getting infected and further spreading the virus (Nejatidanesh et al. 2013).

Transmission of COVID-19 most commonly occurs when an infected patient sneezes, coughs, or even talks, then droplet from his mouth/nose is inhaled by another individual, which indicates that saliva can transmit the disease as it was reported by 97.2% of the participants.

The current study found that a large number of students agree that COVID-19 is a highly dangerous virus (96.9%) that was in accordance with Sengupta et al. (2020) who reported that 95% of the dentists from different countries reported COVID-19 as fatal disease.

Nonetheless, it was found that Egyptian dental students in this research were aware about the main symptoms of COVID-19, which helps the students to recognize the threat and take the necessary precautions, during their future dental practice, which is considered a fundamental part in the management and control of the spread of the virus (Meng et al. 2020; Gaffar et al. 2019).

It was reported that half of participants thought that it is vital to postpone the dental visit for a suspected infected patient with COVID-19, and these results were in accordance with a study done by Guo et al. (2020) and also in accordance with a research conducted by Ahmed et al. (2020), who reported that most of the dentists were fearful to perform dental treatment for a suspected infected patient.

It was established that droplets, saliva and aerosols are considered the main route for transmission of coronavirus (Ather et al. 2020) and that makes the dentists and healthcare workers at high risk of being infected or transmit the disease to their families or to other patients (Ahmed et al. 2020), and it was noticed that most of the participants (97.2%) agreed that saliva can transmit the disease which reflect their knowledge and awareness about routes of transmission of COVID-19.

In the literature, it has been indicated that aerosols and high-speed hand piece have the potential to spread bacteria and viruses to the dentist and dental staff (Barabari and Moharamzadeh 2020; Shah 2020; Checchi et al. 2020). So, choosing hand instruments or low-speed hand piece by most of the participants for dental management of suspected COVID patient indicated their awareness about the role of aerosols in spreading of the viral infection and that proper dental management for a suspected COVID patient is preferred to be by hand instruments.

Most of the students and interns (72%) had no idea about the anti-retraction valve hand piece. Using of high-speed hand piece with anti-retraction valves is considered one of the most important safety measures that hinders microbial cross-infection between patients, as it prevents the backflow of oral fluids and aspiration of biologicals fluids/microbes into the dental air and water tubes; therefore, cross-contamination of the dental unit is prevented (Peng et al. 2020; Xu et al. 2020; Fallahi et al. 2020).

Using rubber dam during dental treatment of the patients plays an essential role not only in isolation of the operating field but it also decreases the splatters of saliva and blood, which consequently decrease the risk of transmission of the viral infection through saliva (Ather et al. 2020; Xu et al. 2020; Villani et al. 2020).

The majority of the participants agreed that they should use rubber dams and saliva ejectors with low or high volume to reduce droplets and aerosols production during endodontic procedure and pulp therapy for children. So, this indicates that the information related to the way of contamination and spreading of COVID-19 and the measures for protection are well known (Peng et al. 2020; Wang et al. 2016; Bizzoca et al. 2020).

All the dental clinics/settings should be cleaned and disinfected as per the applicable regulatory standards, after each patient (Andersen et al. 2006; Gurzawska-Comis et al. 2020); all the work surfaces should be efficiently cleaned and decontaminated with ethyl alcohol (70%). If blood is visible on a surface, it is necessary to use sodium hypochlorite (0.5%) in cleaning and disinfecting the surfaces. It is essential to use protective gloves during sanitization and disinfection. All the procedures should be carried out with care to avoid contact of the disinfecting agents with the skin, eyes or mucosa (Jamal et al. 2020).

One of the most essential steps in preserving the safety and health of medical professionals, healthcare workers, including dentists, is the presence of effective infection control measures, and persistent use of convenient levels of personal protective equipment (PPE) (Khader et al. 2020).

Personal protective equipment (PPE) refers to wearable equipment that is designed to provide protection from exposure to or contact with infectious agents. PPE that is relevant to various types of patient interactions and efficiently covers personal clothing and skin prone to be exposed to saliva, blood or other potentially infectious materials should be used. These include gloves, face masks, protective eye wear, face shields, and protective clothing (e.g., reusable or disposable gown) (Odeh et al. 2020).

The students’ response in favor of personal protective equipment showed that (98.6%) recommended using face shields to provide protection from any blood or saliva splatters and (96.3%) chose disposable gowns as one of the most important personal protective equipment. Surgical masks are used to protect the nose and mouth from the large particles containing pathogens (such as droplets, sprays or splashes). It was also found that surgical masks do not create adequate seal against the skin of the face and therefore are not recommended to protect people from airborne infectious diseases and that clarify the preference of 88.4% of the participants for the N95 mask over the surgical mask (Bizzoca et al. 2020).

Concerning the limitations, being a cross-sectional study, so it recorded the knowledge and awareness of the dental students at the time of the survey and could not follow the improvement of knowledge and awareness of the dental students, that occurs with time.

Regarding the strengths, the results of the current study can be considered as a baseline record which facilitates further evaluation of the improvement of knowledge and awareness of the dental students about COVID-19 and the necessary infection control measures required during performing dental treatment.

## Conclusions

Dental students and inters have good knowledge and awareness about COVID-19 and the necessary precautions required to provide adequate dental treatment for the patients during the pandemic COVID-19; however, the importance of infection control should be highlighted for both clinical and preclinical dental students, to provide safe dental treatment to the patients as well as protection of the dentists and healthcare workers.

## Data Availability

All the data included in the current study are available.

## Abbreviations

PPE: Personal protective equipment
WHO: World Health Organization
SS: Single strand
RR: Ribonucleic acid
CT: Computed tomography
HBV: Hepatitis B virus
HCV: Hepatitis C virus
HIV: Human immunodeficiency virus
AIDS: Acquired immune deficiency syndrome
ADA: American Dental Association
